# Potential dosimetric benefits of adaptive tumor tracking over the internal target volume concept for stereotactic body radiation therapy of pancreatic cancer

**DOI:** 10.1186/s13014-017-0906-9

**Published:** 2017-11-09

**Authors:** Konstantina Karava, Stefanie Ehrbar, Oliver Riesterer, Johannes Roesch, Stefan Glatz, Stephan Klöck, Matthias Guckenberger, Stephanie Tanadini-Lang

**Affiliations:** 1 Department of Radiation Oncology, University Hospital Zurich (USZ), Rämistrasse 100, Zurich, 8091 Switzerland; 2 University of Zurich, Rämistrasse 71, Zurich, 8006 Switzerland

**Keywords:** Radiotherapy, Pancreas, Tumor tracking, Motion management, Stereotactic body radiation therapy

## Abstract

**Background:**

Radiotherapy for pancreatic cancer has two major challenges: (I) the tumor is adjacent to several critical organs and, (II) the mobility of both, the tumor and its surrounding organs at risk (OARs). A treatment planning study simulating stereotactic body radiation therapy (SBRT) for pancreatic tumors with both the internal target volume (ITV) concept and the tumor tracking approach was performed. The two respiratory motion-management techniques were compared in terms of doses to the target volume and organs at risk.

**Methods and Materials:**

Two volumetric-modulated arc therapy (VMAT) treatment plans (5 × 5 Gy) were created for each of the 12 previously treated pancreatic cancer patients, one using the ITV concept and one the tumor tracking approach. To better evaluate the overall dose delivered to the moving tumor volume, 4D dose calculations were performed on four-dimensional computed tomography (4DCT) scans. The resulting planning target volume (PTV) size for each technique was analyzed. Target and OAR dose parameters were reported and analyzed for both 3D and 4D dose calculation.

**Results:**

Tumor motion ranged from 1.3 to 11.2 mm. Tracking led to a reduction of PTV size (max. 39.2%) accompanied with significant better tumor coverage (*p*<0.05, paired Wilcoxon signed rank test) both in 3D and 4D dose calculations and improved organ at risk sparing. Especially for duodenum, stomach and liver, the mean dose was significantly reduced (*p*<0.05) with tracking for 3D and 4D dose calculations.

**Conclusions:**

By using an adaptive tumor tracking approach for respiratory-induced pancreatic motion management, a significant reduction in PTV size can be achieved, which subsequently facilitates treatment planning, and improves organ dose sparing. The dosimetric benefit of tumor tracking is organ and patient-specific.

## Background

Pancreatic cancer is one of the most aggressive tumors with high mortality rates. According to cancer mortality statistics, for 2015 in European Union (EU) a total of 85300 pancreatic cancer deaths are predicted (42700 men and 42600 women), making it the fourth leading cause of cancer-related deaths for both genders in the EU [1].

Surgery is generally considered the standard treatment for patients with resectable pancreatic cancer (PC) while standard-dose chemoradiation therapy and chemotherapy alone are suitable in case of unresectable disease [2]. Stereotactic body radiation therapy (SBRT) allows for higher doses per fraction to the tumor than the conventional radiotherapy with sharp dose gradients in a shorter overall treatment time, and better dose sparing of adjacent organs at risk. It has been successful in the treatment of thoracic tumors and has been used for intra-abdominal sites, including pancreas [2–5]. Besides the patient comfort due to significantly shorter treatment times, additional advantages of SBRT include the possibility of being better integrated into multidisciplinary treatment concepts, significantly less bone marrow toxicity and shorter interruption of full-dose chemotherapy [5]. SBRT for pancreatic tumors has been either delivered by traditional linear accelerator [6, 7] or a robotic arm-mounted linear accelerator [6, 8].

Radiotherapy for pancreatic cancer has two major challenges: (i) the tumor is proximal to several critical organs such as duodenum, stomach, small intestine, kidneys, liver and spinal cord, and (ii) the mobility of both the tumor and its surrounding organs at risk (OARs). The duodenum, which often lies immediately adjacent to pancreatic tumors, is most commonly the organ that limits the maximum dose that can be delivered to the target [9].

As radiotherapy treatments become more and more conformal, without a full understanding of the individualized tumor/organ motion, the target volume could be under-dosed while the nearby OARs could be over-dosed, resulting in the degradation of the overall treatment effectiveness and accuracy. Several studies have addressed the important aspect of pancreatic mobility caused by respiration using different imaging modalities such as magnetic resonance imaging (MRI) [9, 10], four-dimensional computed tomography (4DCT) [11–14], cone-beam computed tomography [15], fluoroscopy [16, 17] and ultrasound [18]. They have shown that, on average, the magnitude of pancreatic motion is largest in the cranial-caudal (superior-inferior) direction and the largest reported value among them was 24±16 mm [10].

The implementation of multi-fraction SBRT has demonstrated promising results with favorable tumor control and toxicity rates [19] which might be further improved using advanced motion management strategies which lead to margin reduction and therefore PTV size reduction.

Different strategies have been proposed to account for respiratory motion [20, 21]: active breath-hold techniques [22], abdominal compression techniques [23], gating during end-expiration [24], motion-encompassing methods (Internal Target Volume (ITV) concept) [25], and respiration-synchronized techniques (tumor tracking approach) [25]. Tumor tracking is an advanced motion management strategy, which adjusts dynamically the treatment beam [26–28] or the patient position [29, 30] to the tumor motion.

Currently, most clinics use the ITV concept, which accounts for the whole tumor motion resulting in broad safety margins. However, among the aforementioned motion management methods, the tumor tracking is recognized as an advanced method of managing respiratory motion reducing the size of the planning target volume (PTV). This might lead to improved targeting and better tumor control while minimizes the radiation-induced toxicity to normal tissue. Real-time tracking of the pancreatic target has been mainly performed by a robotic arm-mounted linear accelerator [31] which utilizes prediction and correlation models [32–34] in order to relate the external breathing signals to 3D tumor motion (Cyberknife Synchrony, Saunnyvale, CA).

The aim of this study was to investigate the possible dosimetric advantages of using a tumor tracking approach compared with the ITV concept to account for respiratory-induced pancreatic tumor motion during gantry-based SBRT. For a better estimation of the dose in the moving tumor, we implemented 4D dose calculation of SBRT treatments with the ITV [25, 35] and the tumor tracking approach [25, 36, 37]. This allows to include respiratory motion effects into the evaluation of both motion managements strategies, which has not been done before so far for pancreatic tumors. Additionally, the differences between the 3D and 4D dose calculations were evaluated for both motion management strategies.

## Methods and materials

### Patients and four-dimensional computed tomography data

Four-dimensional computed tomography (4DCT) data, from 12 consecutive patients with pancreatic cancer, was included in this retrospective study. Among them, three had undergone total duodenectomy. Each patient had received a 4DCT scan using the SOMATOM Definition AS Open (Siemens AG, Germany) CT scanner. Scans were acquired under free breathing without coaching and with respiratory monitoring (RPM, Varian Medical Systems, Palo Alto CA). An average CT and ten respiratory phase CTs were reconstructed. For all but two patients amplitude-sorted 4DCT scans had been obtained, while for two patients phase-sorted 4DCT scans. The data sets were transferred to Eclipse Treatment Planning System (Varian Medical Systems, Palo Alto). Slice thickness of the 4DCT scans was 2 mm and a pitch of 0.09 was used.

### Organ and target segmentation

The gross tumor volume (GTV) and multiple abdominal organs were contoured manually by the radiation oncologists of the Radiation Oncology Department of University Hospital Zurich on one respiratory phase of the 4DCT image set. This contouring phase, which is approximately between inhale and exhale, was considered to better approximate the average tumor position during the full breathing cycle [14]. The following abdominal organs were considered as organs at risk (OAR): duodenum, stomach, bowel, left and right kidneys, liver and spinal cord.

Two individual structure sets were generated to simulate treatment planning for dynamic motion compensation with tumor tracking and for the conventional motion encompassing ITV concept. For tracking, the structures on the contouring phase were used with the GTV as primary target volume (*GTV*_*track*_). For the ITV concept, structure propagation was performed to all respiration phases of the 4DCT image set by deformable registration using MIM Maestro (v6.1, MIM Software Inc., Cleveland, OH). The individual structures from all phases were combined to a total structure, which was then used for treatment planning. The motion envelope enclosing all GTVs was defined as ITV. The OARs were also created from their motion envelope over all respiratory 4DCT phases. An ITV-to-PTV margin of 5 mm was added for the *PTV*_*itv*_. For tracking, a fixed 5mm-margin was added to *GTV*_*track*_ to form the *PTV*_*track*_.

### Tumor motion

The center-of-volume motion for GTV at each respiratory phase along the superior-inferior (SI), anterior-posterior (AP), and left-right axes was measured using MIM software. The tumor motion per direction was determined as the maximal displacement. Three dimensional tumor motion vector was defined as: 3D tumor motion= (*LR* ^2^+*AP* ^2^+*SI* ^2^)^1/2^.

### Treatment planning - three dimensional (3D) dose calculation

For both concepts, ITV and tracking, stereotactic body radiotherapy (SBRT) was planned based on the concept-specific structure sets. SBRT was planned using a nominal energy of 10 MV photon beam in flattening-filter-free mode. Two full arcs with collimator rotations of 5° and 355° were used. Treatment plan optimizations for volumetric-modulated arc therapy (VMAT) and dose calculations using the Analytical Anisotropic Algorithm (AAA) 13.6.23 were performed in Eclipse Treatment Planning System. The treatment plans were calculated on the average reconstruction of the 4DCT data set for the ITV concept and on the contouring phase for the tumor tracking concept. The derived dose distribution is referred from now on as 3D dose distribution.

A dose of 25 Gy in 5 fractions was prescribed to the 60%-isodose surrounding the PTV, which is now widely used [6, 8], corresponding to a maximum allowed dose of up to 41.5 Gy (166% of the prescribed dose). Treatment plans were created with more than 95% of the PTV receiving the prescribed dose. A risk adapted prescription similar to the Simultaneous Integrated Protection (SIP) concept of Gkika et al. was used [38]. Parts of the PTV overlapping with OARs received between a minimum of 25 Gy and a maximum of 27.5 Gy. However, in the parts of the PTV not overlapping with OAR, we were aiming for a minimum dose of 27.5 Gy escalating the dose in the GTV as high as 41.5 Gy. The constraints for OARs were *D*_*max*_<25 Gy on the spinal cord, *D*_*max*_<27.5 Gy for the bowel, the duodenum, the stomach and *D*_*mean*_<10 Gy for the kidneys.

### Four dimensional (4D) dose calculation

Inter-fractional and intra-fractional anatomical variations can introduce significant errors in dose delivered by radiation therapy. The impact of respiratory motion should not be ignored as respiration can induce both rigid body translation/rotation and organ deformation. The 3D dose distribution does not fully represent the dose accumulated by a moving tumor [25]. 4D dose calculation considers the changing beam aperture and the changing anatomy. It strongly depends on the patient’s respiratory cycle and therefore should be performed explicitly on a patient-specific base. In this study, for the 4D dose calculation, we used the concept presented by Ehrbar et al. [25].

The respiratory cycles of all the patients were divided into 10 breathing phases, according to the 4DCT. The original treatment plans (for ITV and tumor tracking concept) were divided into angular segments, which were temporally assigned to the breathing phases. This procedure was done by using in-house MATLAB scripts [25, 39].The created sub-plans were imported back to the treatment planning system and calculated on the different breathing phase. For the tumor tracking concept, the sub-plans were calculated on the different phase CT image set while the beam isocenter was shifted according to the current center of volume position for the GTV to simulate adaptive motion compensation. Subsequently, 4D dose accumulation was performed in MIM Software.

The dose accumulation was performed against the initial contouring phase and the other CT phases were registrated to this one phase using deformable image registration. Following, the dose distributions at other phases were summed up to the dose distribution at reference CT to make 4D dose accumulation [39]. The resulting accumulated 4D dose distributions were recorded for the GTV volume and the OARs of the reference phase.

### PTV size and dosimetric comparisons

The two motion-management techniques were compared regarding the resulting PTV size, dose coverage of the tumor, and sparing of organs at risk. The dosimetric comparisons were performed in 3D and 4D. For the 3D dose comparison, the dose parameters were evaluated on the structures used for treatment planning. Dose parameters for the target (*GTV*_*track*_ and ITV, respectively) include the minimum (*D*_*min*_), maximum (*D*_*max*_), mean dose (*D*_*mean*_), *D*_2_, *D*_95_ and *D*_98_ - the dose received by 2, 95 and 98% of the target volume. For OARs, two dose parameters were recorded: the *D*_*mean*_ and the *D*_0.1*cc*_, which corresponds to the dose received by 0.1 cc of the volume. For the comparison of 4D dose distributions, the same dose parameters were evaluated within the structures delineated on the reference phase for the dose accumulation. This means that for both motion-management techniques, the 4D target dose parameters were evaluated on the GTV.

### Statistical analysis

Dosimetric comparisons between ITV concept and tumor tracking concept were performed with a two-sided Wilcoxon paired signed rank test, with p-values < 0.05 considered significant. The degree of association between the differences in dose parameters and the 3D tumor motion was calculated by Spearman’s rank correlation and a significance level of 5% was used (*p*-value < 0.05). Statistical analysis was performed using R v.3.2.4 [40].

## Results

### Tumor motion and PTV size

**Fig. 1:**
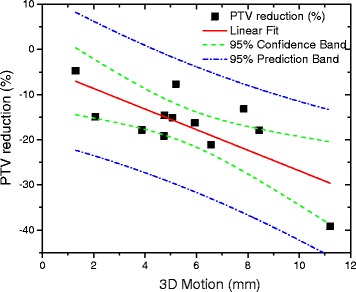
Linear regression between PTV reduction (%) and 3D tumor motion [Equation: y=-2.28*(3D motion)-4.09, *R* ^2^=0.541]

The respiratory-induced pancreatic motion was studied in 12 patients (Table 1). For all patients, the largest amplitude of pancreatic motion was found in the SI direction. The 3D motion ranged from 1.3 to 11.2 mm. With an adaptive tumor tracking approach, the PTV size could be significantly (*p*<0.01) reduced with an average reduction of 16.8% (Table 1). Figure 1 shows the linear regression between PTV reduction and 3D motion. The values are moderately correlated (*rho*=−0.46, *p*=0.131).

**Table 1:** Magnitude of pancreatic motion during respiration and PTV size for ITV and tumor tracking concept

			Respiratory-induced motion amplitude (mm)			
Patient	*PTV*_*ITV*_(*cm* ^3^)	*PTV*_*Tracking*_(*cm* ^3^)	AP	SI	LR	3D Motion
1	54.3	33	2.4	10.4	3.3	11.2
2	146.2	122.4	3.0	5.1	0.6	5.9
3	236.7	200.9	2.0	4.3	1.6	5.1
4	109.8	90.2	1.0	3.7	0.7	3.9
5	154.9	147.6	0.7	1.1	0.3	1.3
6	34.9	28.2	0.5	4.6	1.1	4.7
7	83.0	70.6	1.0	1.6	0.8	2.1
8	114.6	90.4	6.1	1.9	1.7	6.6
9	241.4	209.6	1.8	7.6	1.2	7.8
10	244.0	208.4	1.9	4.3	0.3	4.8
11	177.6	163.9	1.3	5.0	0.5	5.2
12	116.3	95.5	2.1	7.4	3.5	8.5
Median	131.3	109.0	1.9	4.5	1.0	5.2
(prctiles)	(96.4, 207.2)	(80.4, 182.4)	(1.0, 2.3)	(2.8, 6.3)	(0.6, 1.7)	(4.3, 7.2)
Mean	142.8	121.7	2.0	4.8	1.3	5.6
SD	71.2	64.6	1.5	2.7	1.1	2.7

AP = anterior-posterior; SI = superior-inferior; LR = left- right plane; 3D = three dimensional; prctiles = (25-percentile, 75-percentile); SD = standard deviation

### Treatment plan quality

The reduction of PTV size allowed for a decrease of the overlap between the PTV and duodenum. This improved not only OAR dose sparing but also facilitated treatment planning optimization. For five patients (3, 9, 10, 11 and 12) not all the optimization objectives could be satisfied in the ITV treatment plan. For these five cases we had to comprise on the dose to the overlapping part of ITV and OARs (ITV-OAR), where we aimed for a dose of 37.5 Gy to 95% of the overlapping volume. Instead, this volume was compromised to values between 83.5% and 93.9% for these five patients. However, the optimization objectives were fulfilled for all patients using the tumor tracking concept.

### Comparisons of 3D and 4D dose calculations

**Fig. 2:**
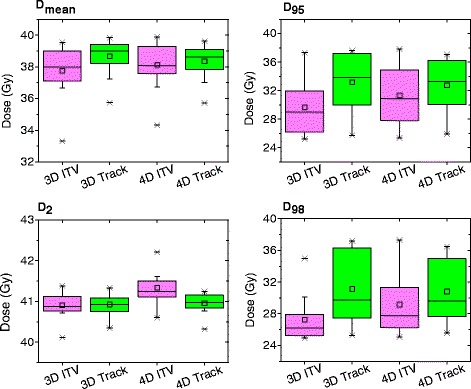
Boxplots indicating the distribution of dose parameters for the target volume (3D & 4D dose) over all patients. Target volume: ITV (ITV concept); GTV (tumor tracking approach). 3D= three dimensional; 4D = four dimensional dose. ITV = internal target volume concept; track = tumor tracking approach

In Table 2, the two motion management techniques are compared using dosimetric parameters of the 3D and 4D dose calculations. The target dose parameters are additionally shown in Fig. 2. There is a clear dosimetric advantage for tumor tracking, regarding the tumor coverage (*D*_95*%*_) in both, 3D and 4D dose calculation. Additionally, many of the OAR dose parameters such as duodenum, stomach, and liver *D*_*mean*_ showed a significant improvement with tracking in 3D and 4D dose. There were also cases in which significant OAR dose reduction was only observed in the 4D dose distribution (bowel, left and right kidney *D*_*mean*_), or the 3D dose distribution (bowel *D*_0.1*cc*_). Furthermore, highly correlated (rho ≥ 0.71), in absolute values, and significant (*p*<0.05) relationships were observed between the 3D dose difference (3*D*_*Tracking*_−3*D*_*ITV*_) and the 3D tumor motion for the bowel *D*_*mean*_ (*rho*=−0.73) and the target volume Dmean (rho = 0.73). No significant results were found for the correlation coefficients investigating the 4D dose differences.

**Table 2:** Comparison of the ITV and tracking concept

Dp [Gy]	Median 3*D*_*ITV*_ [Gy]	Median (prctiles) [Gy] 3*D*_*Tracking*_−3*D*_*ITV*_	*p*-value		Median 4*D*_*ITV*_ [Gy]	Median (prctiles) [Gy] 4*D*_*Tracking*_−4*D*_*ITV*_	*p*-value
	TV				GTV		
*D*_*mean*_	38.00	0.94 (0.39 to 1.09)	< 0.05		38.08	0.27 (–0.16 to 0.55)	0.233
*D*_*max*_	41.55	0.01 (–0.13 to 0.04)	0.791		41.85	–0.27 (–0.98 to 0.14)	0.064
*D*_*min*_	24.59	0.75 (–0.07 to 1.98)	< 0.05		24.83	0.34 (0.19 to 2.00)	0.064
*D*_95_	29.02	3.70 (1.19 to 5.51)	< 0.05		30.87	1.56 (0.65 to 2.43)	< 0.05
*D*_2_	40.87	0.05 (–0.11 to 0.20)	0.677		41.25	-0.27 (-0.81 to -0.04)	< 0.05
*D*_98_	26.20	3.45 (1.24 to 5.30)	< 0.05		27.78	1.73 (0.77 to 2.25)	< 0.05
Bowel							
*D*_*mean*_	4.42	–0.27 (–0.56 to –0.06)	0.092		5.01	–0.45 (–0.58 to –0.24)	< 0.05
*D*_0.1*cc*_	26.87	–1.34 (-1.51 to –0.73)	< 0.05		26.20	–0.60 (–1.14 to 1.11)	0.791
Duodenum							
*D*_*mean*_	9.95	–1.13 (–1.53 to –0.93)	< 0.05		10.45	–1.30 (–1.59 to 0.62)	< 0.05
*D*_0.1*cc*_	27.31	–0.43 (–0.82 to –0.03)	0.164		27.97	0.46 (–1.41 to 0.63)	0.910
Stomach							
*D*_*mean*_	3.34	–0.39 (–0.80 to –0.08)	< 0.05		3.38	–0.30 (–0.60 to –0.07)	< 0.05
*D*_0.1*cc*_	23.57	–1.04 (–2.17 to –0.03)	< 0.05		22.62	–0.11 (–1.11 to 0.97)	0.791
Liver							
*D*_*mean*_	2.04	–0.17 (–0.37 to -0.12)	< 0.05		2.15	–0.18 (–0.35 to -0.07)	< 0.05
Left Kidney							
*D*_*mean*_	3.89	–0.21 (–0.60 to 0.18)	0.266		3.58	–0.29 (–0.60 to -0.15)	< 0.05
Right Kidney							
*D*_*mean*_	3.90	–0.24 (–0.46 to 0.16)	0.176		4.00	–0.40 (–0.57 to 0.12)	< 0.05
Spinal Cord							
*D*_*max*_	9.24	–0.31 (–1.10 to 0.47)	0.569		9.26	–0.37 (–1.55 to 0.56)	0.569

Dp: Dose parameter, prctiles = (25-percentile, 75-percentile), Target volume (TV): (ITV concept); GTV (tumor tracking).

Differences of the dose parameters for tumor volume and organs at risk after 3D and 4D dose calculation are given

**Fig. 3:**
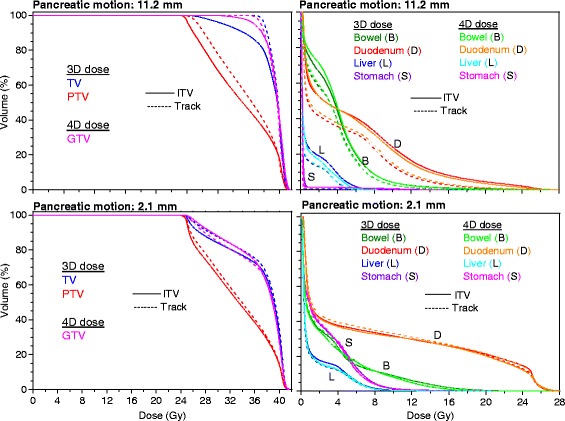
Dose-volume histograms (DVHs) for the target volume (TV), PTV and GTV, and also for the organs at risk (bowel, duodenum, liver, and stomach) for both dose distributions (3D and 4D) and motion management methods (the ITV concept and tumor tracking approach) in case of patient 1 (pancreatic motion: 11.2 mm) and patient 7 (2.1 mm). Target volume: ITV (ITV concept); GTV (tumor tracking approach). 3D = three dimensional; 4D = four dimensional dose. ITV = internal target volume concept; track = tumor tracking approach

Figure 3 shows dose-volume histograms (DVHs) for 3D and 4D dose calculations. The data is presented for two typical patients, patient 1 with pancreatic motion 11.2 mm and patient 7 with pancreatic motion 2.1 mm. For patient 1 there is an obvious gain in the dose coverage of the tumor and OAR dose sparing with tumor tracking approach. However, for patient 7 minimal improvements were observed regarding the tumor coverage and the OAR dose reduction. Additionally, using the ITV concept for patient 1 the 3D dose results in an overestimation for all OARs in comparison with 4D dose calculation. On the other hand, for the same patient using the tumor tracking approach almost no difference between the two dose calculations is noticed with the exception for the dose to the duodenum.

## Discussion

In the present study we investigated the potential dosimetric benefits of using the tumor tracking concept instead of the ITV concept for the respiratory-induced motion of pancreatic tumors. Tumor motion ranged between 2 and 11 mm. Planning target volumes were reduced using the tracking approach with increasing tumor motion. For tumor tracking all 3D plans fulfilled the constraints whereas for the ITV concept we had to compromise the PTV coverage in five cases. Mean and maximum doses to most organs at risk were significantly reduced using tumor tracking. However, using 4D calculation this significant reduction could only be confirmed for the mean doses to the organs at risk.

Our motion results have shown that the magnitude of motion is largest in the SI direction and are in agreement with previous studies with 4DCT. Tai et al. [41], Lens et al. [42], and Hallman et al. [14] found a mean tumor motion between 5 and 8 mm in SI direction. However, in the literature larger pancreas motion has been also reported. Particularly, Minn et al. [43] calculated on a planning 4DCT the range of centroid movement in SI direction between 0.9 to 28.8 m (from the maximum inspiration to the maximum expiration). Similar large motion amplitudes were reported by Bussels et al. [10] and Feng et al. [9] using MRI imaging. Observed differences in the pancreatic tumor motion between the current work and the previous studies may be due to patient heterogeneity and variation of inter-fractional pancreatic motion [41].

When the ITV concept is used, a large volume is unnecessarily irradiated since the whole envelope of motion is treated. This can be avoided if the tumor tracking is considered. Our findings with the tumor tracking approach led to a mean PTV volume reduction of 16.8*%*±8.4*%* (range: −4.7 to −39.2*%*). This significant reduction of the PTV is in agreement with the result found by Lens et al.[44] using the midventilation approach as a motion management technique for the pancreatic tumors. They found a mean absolute PTV volume reduction of 13.9%. However, it is worth mentioning that this single-phase tracking is able to deposit higher dose to target volume than the midventilation approach.

Tumor tracking allowed fulfilling all planning objectives whereas for five ITV plans we had to make a compromise on the PTV coverage in order to spare the organs at risk adequately. Therefore, we consider tracking as a promising tool for complicated cases for which the objectives cannot be fulfilled using the ITV concept. Similarly, for tracking of lung tumors Ehrbar et al. [25] showed a significant reduction in the mean dose to the lung compared to the ITV concept.

The 4D dose calculation can be considered as an appropriate tool for better understanding of the differences between a static 3D dose distribution and taking the respiratory motion into account. 4D dose calculation has been also performed by other authors [25, 35–37, 45] but mostly for lung [25, 37, 45] and not for pancreas. The results of our study have shown that the 3D dose distribution adequately represented the actual 4D dose for the mean tumor dose as well as the coverage of the tumor for both concepts. This is different compared to investigations for the lung, where the 3D dose calculation underestimates the dose to the tumor compared to the actual 4D calculation for the ITV concept [25, 45]. This difference is due to the changing density in the lung, which is not that pronounced in the pancreatic region.

In our study a single 4DCT was acquired per patient and based on this, we performed our treatment planning. However, it has been shown that the respiratory-induced tumor motion in pancreatic cancer patients given by a single 4DCT is not necessarily representative of the amplitude during treatment [42]. Furthermore, during the treatment of pancreatic tumors, non-rigid shape and position variations occur frequently both in the tumor and the OARs [31]. These effects were not considered in this work as structure delineation and 4D dose calculations were based on a single 4DCT before the treatment. Respiratory patterns reproducibility during the imaging and delivery is an important issue that needs further investigation [20]. Such changing breathing and motion patterns are not an issue for adaptive tracking approaches which use real-time position feedback of the tumor, since they are able to adapt to the actual tumor position during delivery and this improves the daily both tumor coverage and OAR sparing.

Another limitation of the study was that we assumed perfect tumor tracking. We wanted to show what is possible with a perfect system rather than evaluating possible current systems. We assumed that generating the PTV with a 5 mm safety margin on the ITV/GTV, may be adequate to account for tracking errors, residual organ deformation and rotation [46]. However, we performed 4D dose calculation which deals with the main contributors of tracking inaccuracies such as rotation and deformation [47].

Our results indicate that there is a possible benefit of using tumor tracking approach instead of ITV concept in SBRT treatment for pancreatic cancer patients. However, further investigation is needed in order to relate the present results to clinical outcome in terms of toxicity. This study showed that the tumor motion differs between patients, indicating that tumor motion should be assessed individually. Furthermore, the tumor tracking approach resulted in better tumor coverage and a significant reduction of PTV facilitating subsequently treatment planning and decreasing mean doses of OARs.

## Conclusions

Taking the pancreatic tumor motion into account is important and critical in SBRT. If this movement is neglected, it could offset the potential benefits of SBRT. In this study two motion management techniques, ITV concept and tumor tracking concept, were evaluated and compared to each other using 3D and 4D dose calculations.

The dosimetric comparisons showed that the dose benefit is patient and OAR specific. Tumor tracking might be considered for patients in whom the planning objectives with ITV concept cannot be met. However, tumor tracking requires a 4D dose calculation to have an accurate estimation of the doses to the organs at risk and additionally, a suitable position information and motion compensation system.

## Abbreviations

3D: Three-dimensional
4D: Four-dimensional
4DCT: Four-dimensional computed tomography
AAA: Analytical anisotropic algorithm
AP: Anterior - posterior
D_2_: Dose received by the 2% of target volume
D_95_: Dose received by the 2% of target volume
D_98_: Dose received by the 2% of target volume
D _*max*_: Maximum dose
D _*mean*_: Mean dose
D _*min*_: Minimum dose
DVH: Dose-volume histograms
EU: European Union
GTV: Gross tumor volume
ITV: Internal target volume
MRI: Magnetic resonance imaging
OAR: Organ at risk
PC: Pancreatic cancer
PTV: Planning target volume
SBRT: Stereotactic body radiation therapy
SI: Superior - inferior
SIP: Simultaneous integrated protection
TV: Target volume
VMAT: Volumetric-modulated arc therapy

## References

[1] Malvezzi M, Bertuccio P, Rosso T, Rota M, Levi F, La Vecchia C, Negri E (2015). European cancer mortality predictions for the year 2015: does lung cancer have the highest death rate in EU women?. Ann Oncol.

[2] Petrelli F, Comito T, Ghidini A, Torri V, Scorsetti M, Barni S (2017). Stereotactic body radiation therapy for locally advanced pancreatic cancer: a systematic review and pooled analysis of 19 trials. Int J Radiat Oncol Biol Phys.

[3] Trakul N, Koong AC, Chang DT (2014). Stereotactic body radiotherapy in the treatment of pancreatic cancer. Semin Radiat Oncol.

[4] Casamassima F, Cavedon C, Francescon P, Stancanello J, Avanzo M, Cora S (2006). Use of motion tracking in stereotactic body radiotherapy: Evaluation of uncertainty in off-target dose distribution and optimization strategies. Acta Oncol.

[5] Panje C, Andratschke N, Brunner TB, Niyazi M, Guckenberger M (2016). Stereotactic body radiotherapy for renal cell cancer and pancreatic cancer: literature review and practice recommendations of the DEGRO working group on stereotactic radiotherapy. Strahlenther Onkol.

[6] Pollom EL, Alagappan M, von Eyben R, Kunz PL, Fisher GA, Ford JA (2014). Single- versus multifraction stereotactic body radiation therapy for pancreatic adenocarcinoma: outcomes and toxicity. Int J Radiat Oncol Biol Phys.

[7] Mellon EA, Hoffe SE, Springett GM, Frakes JM, Strom TJ, Hodul PJ (2015). Long-term outcomes of induction chemotherapy and neoadjuvant stereotactic body radiotherapy for borderline resectable and locally advanced pancreatic adenocarcinoma. Acta Oncol.

[8] Chuong MD, Springett GM, Freilich JM, Park CK, Weber JM, Mellon EA (2013). Stereotactic body radiation therapy for locally advanced and borderline resectable pancreatic cancer is effective and well tolerated. Int J Radiat Oncol Biol Phys.

[9] Feng M, Balter JM, Normolle D, Adusumili S, Cao Y, Chenevert TL (2009). Characterization of pancreatic tumor motion using cine MRI: surrogates for tumor position should be used with caution. Int J Radiat Oncol Biol Phys.

[10] Bussels B, Goethals L, Feron M, Bielen D, Dymarkowski S, Suetens P (2003). Respiration-induced movement of the upper abdominal organs: a pitfall for the three-dimensional conformal radiation treatment of pancreatic cancer. Radiother Oncol.

[11] Goldstein SD, Ford EC, Duhon M, McNutt T, Wong J, Herman JM (2010). Use of respiratory-correlated four-dimensional computed tomography to determine acceptable treatment margins for locally advanced pancreatic adenocarcinoma. Int J Radiat Oncol Biol Phys.

[12] Cattaneo GM, Passoni P, Sangalli G, Slim N, Longobardi B, Mancosu P (2010). Internal target volume defined by contrast-enhanced 4D-CT scan in unresectable pancreatic tumour: Evaluation and reproducibility. Radiother Oncol.

[13] Shiinoki T, Shibuya K, Nakamura M, Nakamura A, Matsuo Y, Sawada A (2011). Interfractional reproducibility in pancreatic position based on four-dimensional computed tomography. Radiat Oncol Biol Phys.

[14] Hallman JL, Mori S, Sharp GC, Lu HM, Hong TS, Chen GT (2012). A four-dimensional computed tomography analysis of multiorgan abdominal motion. Int J Radiat Oncol Biol Phys.

[15] Ohira S, Isono M, Ueda Y, Hirata T, Ashida R, Takahashi H (2017). Assessment with cone-beam computed tomography of intrafractional motion and interfractional position changes of resectable and borderline resectable pancreatic tumours with implanted fiducial marker. Br J Radiol.

[16] Gierga DP, Chen GT, Kung JH, Betke M, Lombardi J, Willett CG (2004). Quantification of respiration-induced abdominal tumor motion and its impact on imrt dose distributions. Int J Radiat Oncol Biol Phys.

[17] Bhasin DK, Rana SS, Jahagirdar S, Nagi B (2006). Does the pancreas move with respiration?. Gastroenterol Hepatol.

[18] Suramo I, Päivänsalo M, Myllylä V (1984). Cranio-caudal movements of the liver, pancreas and kidneys in respiration. Acta Radiol Diagn (Stockh).

[19] Moningi S, Marciscano AE, Rosati L, Sook Ng K, Teboh Forbang R, Jackson J (2014). Stereotactic body radiation therapy in pancreatic cancer: the new frontier. Expert Rev Anticancer Ther.

[20] Keall PJ, Mageras GS, Balter JM, Emery RS, Forster KM, Jiang SB (2006). The management of respiratory motion in radiation oncology report of AAPM task group 76. Med Phys.

[21] Seuntjens J, Lartigau EF, Cora S, Ding GX, Goetsch S, Nuyttens J (2014). Prescribing, recording, and reporting of stereotactic treatments with small photon beams. J ICRU.

[22] Dawson LA, Brock KK, Kazanjian S, Fitch D, McGinn CJ, Lawrence TS (2001). The reproducibility of organ position using active breathing control (ABC) during liver radiotherapy. Int J Radiat Oncol Biol Phys.

[23] Murray B, Forster K, Timmerman R (2007). Frame-based immobilization and targeting for stereotactic body radiation therapy. Med Dosim.

[24] Taniguchi CM, Murphy JD, Eclov N, Atwood TF, Kielar KN, Christman-Skieller C (2013). Dosimetric analysis of organs at risk during expiratory gating in stereotactic body radiation therapy for pancreatic cancer. Int J Radiat Oncol Biol Phys.

[25] Ehrbar S, Jöhl A, Tartas A, Stark LS, Riesterer O, Klöck S (2017). Itv, mid-ventilation, gating or couch tracking - a comparison of respiratory motion-management techniques based on 4D dose calculations. Radiother Oncol.

[26] Kilby W, Dooley JR, Kuduvalli G, Sayeh S, Maurer C R J (2010). The CyberKnife robotic radiosurgery system in 2010. Technol Cancer Res Treat.

[27] Depuydt T, Verellen D, Haas O, Gevaert T, Linthout N, Duchateau M (2011). Geometric accuracy of a novel gimbals based radiation therapy tumor tracking system. Radiother Oncol.

[28] Keall PJ, Colvill E, O’Brien R, Ng JA, Poulsen PR, Eade T (2014). The first clinical implementation of electromagnetic transponder-guided MLC tracking. Med Phys.

[29] Lang S, Zeimetz J, Ochsner G, Schmid Daners M, Riesterer O, Klöck S (2014). Development and evaluation of a prototype tracking system using the treatment couch. Med Phys.

[30] D’Souza WD, Naqvi SA, Yu CX (2005). Real-time intra-fraction-motion tracking using the treatment couch: a feasibility study. Phys med Biol.

[31] Papalazarou C, Klop GJ, Milder MTW, Marijnissen JPA, Gupta V, Heijmen BJM (2017). CyberKnife with integrated CT-on-rails: System description and first clinical application for pancreas SBRT. Med Phys.

[32] Malinowski K, McAvoy TJ, George R, Dieterich S, D’Souza WD (2012). Online monitoring and error detection of real-time tumor displacement prediction accuracy using control limits on respiratory surrogate statistics. Med Phys.

[33] Malinowski K, McAvoy TJ, George R, Dieterich S, D’Souza WD (2012). Incidence of changes in respiration-induced tumor motion and its relationship with respiratory surrogates during individual treatment fractions. Int J Radiat Oncol Biol Phys.

[34] Zhang H, Zhao G, Djajaputra D, Xie Y (2014). Determination of acquisition frequency for intrafractional motion of pancreas in CyberKnife radiotherapy. Sci World J.

[35] Rietzel E, Chen GT, Choi NC, Willet CG (2005). Four-dimensional image-based treatment planning: target volume segmentation and dose calculation in the presence of respiratory motion. Int J Radiat Oncol Biol Phys.

[36] Schlaefer A, Fisseler J, Dieterich S, Shiomi H, Cleary K, Schweikard A (2005). Feasibility of four-dimensional conformal planning for robotic radiosurgery. Med Phys.

[37] Chan MK, Kwong DL, Ng SC, Tam EK, Tong AS (2012). Investigation of four-dimensional (4D) Monte Carlo dose calculation in real-time tumor tracking stereotactic body radiotherapy for lung cancers. Med Phys.

[38] Gkika E, Adebahr S, Kirste S, Schimek-Jasch T, Wiehle R, Claus R (2017). Stereotactic body radiotherapy (SBRT) in recurrent or oligometastatic pancreatic cancer: A toxicity review of simultaneous integrated protection (SIP) versus conventional SBRT. Strahlenther Onkol.

[39] Ehrbar S, Lang S, Stieb S, Riesterer O, Stark LS, Guckenberger M (2016). Three-dimensional versus four-dimensional dose calculation for volumetric modulated arc therapy of hypo-fractionated treatments. J Med Phys.

[40] R Development Core Team. R: A language and environment for statistical computing. Vienna; 2017. https://www.r-project.org.

[41] Tai A, Liang Z, Erickson B, Li XA (2013). Management of respiration-induced motion with 4-dimensional computed tomography (4DCT) for pancreas irradiation. Int J Radiat Oncol Biol Phys.

[42] Lens E, Van der Horst A, Kroon PS, van Hooft JE, Fajardo RD, Fockens P (2014). Differences in respiratory induced pancreatic tumor motion between 4D treatment planning CT and daily cone beam CT, measured using intratumoral fiducials. Acta Oncol.

[43] Minn AY, Schellenberg D, Maxim P, Suh Y, McKenna S, Cox B (2009). Pancreatic tumor motion on a single planning 4D-CT does not correlate with intrafraction tumor motion during treatment. Am J Clin Oncol.

[44] Lens E, van der Horst A, Versteijne E, van Tienhoven G, Bel A (2015). Dosimetric advantages of midventilation compared with internal target volume for radiation therapy of pancreatic cancer. Int J Radiat Oncol Biol Phys.

[45] Guckenberger M, Wilbert J, Krieger T, Richter A, Baier K, Meyer J (2007). Four-dimensional treatment planning for stereotactic body radiotherapy. Int J Radiat Oncol Biol Phys.

[46] Chan M, Grehn M, Cremers F, Siebert FA, Wurster S, Huttenlocher S (2017). Dosimetric implications of residual tracking errors during robotic SBRT of liver metastases. Int J Radiat Oncol Biol Phys.

[47] Chan MK, Werner R, Ayadi M, Blanck O (2015). Comparison of 3D and 4D Monte Carlo optimization in robotic tracking stereotactic body radiotherapy of lung cancer. Strahlenther Onkol.

